# Acute and late toxicity in prostate cancer patients treated by dose escalated intensity modulated radiation therapy and organ tracking

**DOI:** 10.1186/1748-717X-3-35

**Published:** 2008-10-20

**Authors:** Pirus Ghadjar, Jacqueline Vock, Daniel Vetterli, Peter Manser, Roland Bigler, Jan Tille, Axel Madlung, Frank Behrensmeier, Roberto Mini, Daniel M Aebersold

**Affiliations:** 1 Department of Radiation Oncology with Division of Medical Radiation Physics, University of Bern, Inselspital, Bern, Switzerland, Freiburgstrasse, 3010 Bern, Switzerland

## Abstract

**Background:**

To report acute and late toxicity in prostate cancer patients treated by dose escalated intensity-modulated radiation therapy (IMRT) and organ tracking.

**Methods:**

From 06/2004 to 12/2005 39 men were treated by 80 Gy IMRT along with organ tracking. Median age was 69 years, risk of recurrence was low 18%, intermediate 21% and high in 61% patients. Hormone therapy (HT) was received by 74% of patients. Toxicity was scored according to the CTC scale version 3.0. Median follow-up (FU) was 29 months.

**Results:**

Acute and maximal late grade 2 gastrointestinal (GI) toxicity was 3% and 8%, late grade 2 GI toxicity dropped to 0% at the end of FU. No acute or late grade 3 GI toxicity was observed. Grade 2 and 3 pre-treatment genitourinary (GU) morbidity (PGUM) was 20% and 5%. Acute and maximal late grade 2 GU toxicity was 56% and 28% and late grade 2 GU toxicity decreased to 15% of patients at the end of FU. Acute and maximal late grade 3 GU toxicity was 8% and 3%, respectively. Decreased late ≥ grade 2 GU toxicity free survival was associated with higher age (P = .025), absence of HT (P = .016) and higher PGUM (P < .001).

**Discussion:**

GI toxicity rates after IMRT and organ tracking are excellent, GU toxicity rates are strongly related to PGUM.

## Background

Prostate cancer (PCA) is the most commonly diagnosed cancer among men in Europe and North America [1,2] after skin cancer. In the last decade significant improvement in planning and delivery of radiotherapy (RT) such as three-dimensional conformal RT (3D-CRT) and intensity-modulated radiation therapy (IMRT) have been developed [3-5] allowing the delivery of higher radiation doses. Dose escalated RT has been shown to improve biochemical control rates in PCA, however, to date, this has not translated into better overall survival [5-12]. Dose escalated 3D-CRT has led to increased gastrointestinal (GI) toxicity [10,13,14]. High-dose RT for prostate cancer delivered by IMRT causes lower acute and late GI toxicity rates as compared to 3D-CRT [4] and improvement in tumor delineation such as matching of an magnetic resonance imaging (MRI) of the pelvis with the planning computed tomography (CT) has been shown to reduce GI toxicity in dose escalated RT due to decreased dose to the rectal wall [15].

On the other hand localization of the prostate by image guided radiation therapy (IGRT) is a reasonable approach to reduce toxicity in dose escalated RT of PCA, taking into account the interfractional variability of the prostate position. Therefore we treated prostate cancer patients with dose escalated high precision RT by IMRT along with organ tracking, to reduce toxicity, especially of the GI tract. This work presents the pre-treatment GI and genitourinary (GU) morbidity and acute and late toxicity of the first 39 patients after a median follow-up of 29 months.

## Methods

### Patient selection and characteristics

A total of 46 consecutive men with histologically proven adenocarcinoma of the prostate and cM0 stage were treated by IMRT along with organ tracking after providing informed consent in accordance with the standards of the local ethics committee and with the Helsinki Declaration of 1975, as revised in 1983. The pre-treatment staging included a complete history, physical examination, digital rectal examination, transrectal ultrasound (TRUS) of the prostate, biopsy with specification of the Gleason score, prostate-specific antigen (PSA) level, CT and/or MRI of the abdomen and pelvis and a total-body bone scan. When clinically indicated a chest x-ray and/or thoracic CT was performed. Based on these examinations clinical staging was defined according to the 2002 American Joint Committee on Cancer tumor, lymph nodes and metastasis system [16]. Patients were grouped according to their risk of recurrence as recommended by the National Comprehensive Cancer Network practise guidelines in oncology . Patients with multiple adverse factors were not shifted to the next higher risk group. Seven patients with previous implantation of hip prosthesis (n = 3) with changing of the treatment plan during the treatment (n = 1) and with different dose constraints than described below (n = 1) were excluded from the study. One patient died because of pancreatic cancer < 2 months after RT and one patient died because of bile duct cancer < 9 months after RT, both were also excluded because of insufficient follow-up. The study population thus consisted of 39 patients, treated between 06/2004 and 12/2005, who were retrospectively analyzed. All patients were Caucasian and no patient had received previous pelvic RT. All patients completed the planned course of radiation, receiving treatment as outlined below. Median age of the patients was 69 years (range, 54 – 81 years), median follow-up was 29 months (range, 22 – 40 months). Risk of recurrence was low in 7 (18%), intermediate in 8 (21%) or high in 24 (61%) patients.

### Hormonal therapy

A total of 29 patients (74%) received hormonal therapy (HT), either neoadjuvant, concomitant or adjuvant, with a median duration of 6 months (range, 2 – 24 months) but always during RT. Twenty-six patients received combined androgen blockade (CAB) consisting of antiandrogen and gonadotropin releasing hormone analogue (GnRH). Three patients received antiandrogen monotherapy. Pre-irradiation HT was given to 26 patients (67%) with a median duration of 1.5 months (range, 0.3 – 3.5 months) before starting RT.

### External beam radiotherapy

All 39 patients included in this study were treated using IMRT with organ tracking as described previously [17]. Briefly, the dose was delivered in daily fractions of 2 Gy, given five sessions per week, to a total of 80 Gy. Prior to the treatment, three gold markers (diameter = 0.9 mm, length = 7 mm) were implanted into the prostate of every patient under TRUS guidance. Thirty six of 39 patients underwent an MRI scan of the pelvis in our radiology department. In these patients, digital data was used for image fusion with the planning CT scan. To produce high-resolution digitally reconstructed radiographs (DRRs), consecutive CT images (GE Prospeed^®^) with an interslice spacing and thickness of 3 mm were obtained. To estimate the risk for seminal vesicle (SV) invasion, the Roach formula [18] was calculated for each patient. If the risk for SV invasion was > 15%, as it was the case in 21 patients, the proximal third of the SV was included to the clinical target volume (CTV) electively. If invasion of the base of the SV was seen in the MRI (cT3b), the whole SV were electively included to the CTV. The planning target volume (PTV) was then delineated by encompassing the prostate with 5 mm safety margins in all directions but dorsal, where a 3 mm margin was added. In addition to the PTV, the walls of the rectum and bladder, the femoral heads and the skin surface were also identified. A coplanar five-field IMRT technique with fields placed at angles of 45°, 90°, 180°, 270°, and 320° was used to treat prostate cancer patients with 15MV X-rays (Clinac 2300C/D, Varian Medical Systems). The dose was applied by the dynamic MLC technique using an 80 leaf-MLC. The 80 Gy were prescribed to the median of the PTV. More than 99% of the PTV volume was included in the 95% isodose and the maximum dose was limited to 105%. To limit the dose to the organs at risk (OAR), a template was used with a set of dose constraints. Daily treatment was intended with a full bladder. The isocenter was placed 7 cm behind the symphysis and at the height of the cranial end of the opening of the foramen obturatorium. Partial DRRs were generated for all treatment beams and in addition for two extra setup beams from the anterior-posterior (AP) and the lateral direction (LAT). The field size of the AP setup field was chosen to encompass the implanted markers with a margin of about 1.5 cm. The LAT field enclosed the markers and the symphysis. With a drawing tool the marker positions were manually highlighted, serving as a layer structure for subsequent portal image evaluation. The two setup DRRs served as a reference for portal images taken before daily treatment. For isocenter verification, the patients were simulated (Ximatron^®^, Varian Medical Systems) using the two additional setup fields. The isocenter was then marked on the patient's skin. In the treatment room, the patients were aligned on a carbon-fiber couch panel within their immobilization device using the skin marks. Before each treatment fraction the two setup fields were acquired using the aS500^® ^portal imager (Varian Medical Systems) with a dose saving acquisition mode. The setup and correction of patients position was performed as previously described [19]. Briefly, setup images from the AP and LAT direction were taken with the 6 MV beam. A comparison between positions of the fiducial markers on the portal image with the corresponding reference DRR followed. Patient position was then manually adjusted in all 3 dimensions until the best center of mass (CM) alignment was found with a CM >1 mm limit in any direction. Following this sequential setup procedure, the patient was treated with the multi-field IMRT plan. Optimization of the IMRT plan included a dose limit within the central periurethral area of 99% of the prescribed dose. The duration of IMRT reached a median of 56 days (range, 52 – 63 days).

### Dose constraints

A maximum dose of 100% to the urethra was allowed. For the rectal wall (from the ischial tuberosity to the bottom of the sacroiliac joint), constraints described by Leibel et al. [5] and Boersma et al. [13] were used: the median V47 (percentage of the rectal wall receiving at least 47 Gy) was set to <53% and the median V75 to <5%. For the bladder the constraint from Leibel et al. was used: the median V47 (percentage of bladder wall receiving at least 47 Gy) was set to <53%.

To assess coverage, homogeneity and conformity of the PTV the V95 (percentage of PTV receiving at least 95% of dose, corresponding to 76 Gy) and the Dmax and Dmin (maximum and minimum dose within the PTV) were calculated from the dose volume histograms.

### Follow-up protocol

Patients were seen by a radiation oncologist at least weekly during the RT. Follow-up visits were arranged 2–4 weeks after completion of IMRT and every 3 to 6 months for the first 2 years and annually thereafter with a digital rectal examination and a serum PSA level obtained at each visit. Patients alternated follow-up visits between their urologist and radiation oncologist. A minority of patients who did not attend these visits were contacted and data were successfully retrieved.

### Toxicity scoring

Toxicity was graded using the common terminology criteria for adverse events (CTC AE) version 3.0  from the National Cancer Institute. Late toxicity was defined as complications occurring three months after the end of treatment. Late toxicity at last follow-up visit was determined and was termed last late toxicity. This was done to assess whether the late toxicity was persistent or was transient.

### Statistical analysis

Descriptives include absolute and relative frequencies for categorical variables, median and range for quantitative variables.

The primary endpoint was the occurrence of acute and late ≥ grade 2 GI and GU toxicity. For statistical analysis age (≤ vs > 69 years), PTV (≤ vs > 105 cm^3^), PTV Dmax (≤ vs > 84.3 Gy) and bladder V47 (≤ vs >28%) were grouped according to the median. Pre treatment GU toxicity (PGUM) (grade 0–1 vs grade 2–3) and acute and late GU toxicity (grade 0–1 vs grade 2–3) were grouped. Acute GU toxicity and PGUM were compared using the Fisher's exact test. Estimation of actuarial rates for late ≥ grade 2 GU toxicity free survival was calculated using the Kaplan-Meier product limit methodology. Kaplan-Meier rates were compared using the log-rank test [20]. Cox regression models were used to determine independent prognostic factors for decreased late ≥ grade 2 GU toxicity free survival [21]. Variables were included to the model if the univariate P-value was <.1, thus, age, HT and PGUM were included. Backward model selection was performed in order to identify predictors for decreased ≥ grade 2 late GU toxicity free survival. Statistical significance was considered on a two-sided level of α = 0.05. Statistical analysis was performed with SPSS version 16.0 (SPSS Inc., Chicago, IL).

## Results

### Coverage, homogeneity and conformity of the PTV

For the PTV, the median V95 was 97.9% (range, 89.2 – 99.8%). Median Dmax and Dmin were 84.3 Gy (range, 82.2–85.4 Gy) and 72.1 Gy (range, 67.5 – 74.6 Gy), respectively.

### Acute and late GI Toxicity

Patient characteristics are summarized in Table 1. The pre-treatment GI morbidity, acute and late GI toxicity and incidence of late GI toxicity at last follow-up visit are summarized in Table 2. Incidence of single symptoms (diarrhea, rectal pain, rectal bleeding) as well as the highest toxicity occurred in a given patient concluding all single symptoms but counting only the highest toxicity as a single event are depicted. Acute grade 2 GI toxicity was 1 (3%) and maximal late grade 2 GI toxicity was 3 (8%). No grade 3 GI toxicity occurred. The median time from the completion of RT to the occurrence of ≥ grade 1 late GI was 14 months (range, 6 – 30 months). Late GI toxicity decreased as time from treatment elapsed. At last follow-up visit the incidence of late grade 2 GI toxicity was 0%. There was no association observed between clinical or dosimetric parameters and acute GI toxicity or late GI toxicity free survival.

**Table 1:** Patient characteristics

**Total patients**		**(n = 39)**
***Age***		
	≤ 69 years	20 (51.3%)
	> 69 years	19 (48.7%)
***Tumor stage***		
	cT1	12 (30.8%)
	cT2	5 (12.8%)
	cT3a	13 (33.4%)
	cT3b	7 (17.9%)
	cT4	2 (5.1%)
***Gleason score***		
	2–6	20 (51.3%)
	7	13 (33.3%)
	8–10	6 (15.4%)
***Pre-treatment PSA***		
	≤ 10 ng/mL	20 (51.3%)
	>10 ng/mL	19 (48.7%)
***Risk group***		
	Low	7 (17.9%)
	Intermediate	8 (20.5%)
	High	24 (61.6%)
***Hormonal therapy***		
	no	10 (25.6%)
	yes	29 (74.4%)
***Median FU months† (range)***		29 (22–40)

Abbreviations: PSA = prostate- specific antigen; FU = follow-up;

†period after completion of treatment.

**Table 2:** Pre-treatment gastrointestinal morbidity and gastrointestinal acute and late toxicity

		**Pre-Tx**	**Acute†**	**Late‡**	**Last late§**
Toxicity	Grade	n (%)	n (%)	n (%)	n (%)

Diarrhea	0	39 (100)	29 (74)	36 (92)	38 (97)
	1	0	9 (23)	3 (8)	1 (3)
	2	0	1 (3)	0	0
Rectal pain	0	38 (97)	34 (87)	37 (96)	38 (97)
	1	1 (3)	5 (13)	1 (3)	1 (3)
	2	0	0	1 (3)	0
Rectal bleeding	0	36 (92)	36 (92)	33 (85)	34 (87)
	1	3 (8)	3 (8)	4 (10)	5 (13)
	2	0	0	2 (5)	0
Highest GI*	0	36 (92)	25 (64)	30 (77)	32 (82)
	1	3 (8)	13 (33)	6 (15)	7 (18)
	2	0	1 (3)	3 (8)	0

Abbreviations: Pre-Tx = pre-treatment; * The highest toxicity in a patient was counted as a single event; † During therapy and until 3 months after completion; ‡ maximal late toxicity > 3 months after completion of therapy; § Incidence of late toxicity at last follow-up visit

### Acute and late GU Toxicity

The PGUM and acute and late GU toxicity are summarized in Table 3. Incidence of single symptoms (dysuria, incontinence, retention, frequency/urgency, hematuria) as well as the highest toxicity occurred in a given patient concluding all single symptoms but counting only the highest toxicity as a single event are depicted. Before treatment 8 patients (20%) had grade 2 and 2 patients (5%) had grade 3 PGUM. After treatment acute grade 2 GU toxicity occurred in 22 (56%) and maximal late grade 2 in 11 (28%) patients. Acute and maximal late grade 3 GU toxicity occurred in 3 (8%) and 1 (3%) patients, respectively. There was no grade 4 GU toxicity observed. The median time from the completion of RT to the occurrence of late ≥ grade 2 GU toxicity was 12 months (range, 4 – 26 months).

**Table 3:** Pre-treatment genitourinary morbidity and genitourinary acute and late toxicity

		**Pre-Tx**	**Acute†**	**Late‡**	**Last late§**
Toxicity	Grade	n (%)	n (%)	n (%)	n (%)

Dysuria	0	36 (92)	20 (51)	35 (90)	38 (97)
	1	3 (8)	13 (33)	4 (10)	1 (3)
	2	0	5 (13)	0	0
	3	0	1 (3)	0	0
Incontinence	0	34 (87)	33 (84)	34 (87)	37 (94)
	1	4 (10)	5 (13)	4 (10)	1 (3)
	2	1 (3)	1 (3)	1 (3)	1 (3)
Retention	0	20 (51)	16 (41)	23 (59)	34 (86)
	1	13 (33)	9 (23)	11 (28)	3 (8)
	2	5 (13)	12 (31)	4 (10)	1 (3)
	3	1 (3)	2 (5)	1 (3)	1 (3)
Frequency/urgency	0	23 (59)	6 (15)	17 (44)	23 (59)
	1	13 (33)	16 (41)	15 (38)	14 (36)
	2	2 (5)	15 (39)	7 (18)	2 (5)
	3	1 (3)	2 (5)	0	0
Hematuria	0	39 (100)	39 (100)	36 (92)	37 (95)
	1	0	0	1 (3)	2 (5)
	2	0	0	2 (5)	0
Highest GU*	0	15 (39)	1 (3)	11 (28)	19 (49)
	1	14 (36)	13 (33)	16 (41)	13 (33)
	2	8 (20)	22 (56)	11 (28)	6 (15)
	3	2 (5)	3 (8)	1 (3)	1 (3)

Abbreviations: Pre-Tx = pre-treatment; * The highest toxicity in a patient was counted as a single event; † During therapy and until 3 months after completion; ‡ maximal late toxicity > 3 months after completion of therapy; § Incidence of late toxicity at last follow-up visit

Interestingly late GU toxicity decreased as time from treatment elapsed. At the last follow-up visit only 6 (15%) of patients had late grade 2 and 1 (3%) had late grade 3 GU toxicity, being fewer patients than initially observed with grade 2 or 3 PGUM. The patient who developed late grade 3 GU toxicity had a bulbar urethral stricture and was treated twice by urethral dilatation, however obstructive symptoms were persistent and ever since the patient uses daily self-catheterism.

### Factors associated with GU toxicity

**Figure 1:**
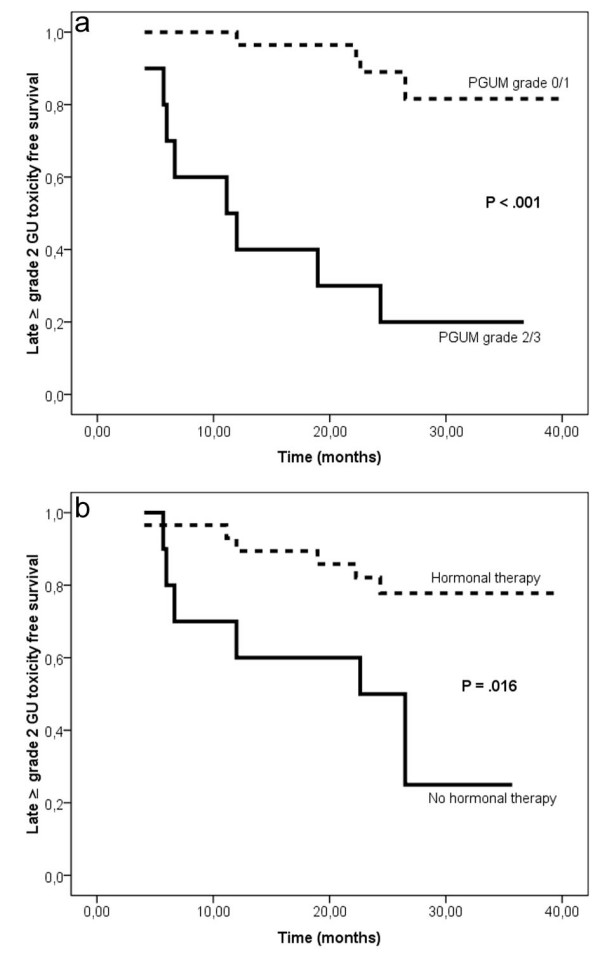
Actuarial analysis of three year late ≥ grade 2 GU toxicity free survival stratified by (a) pre-treatment genitourinary morbidity (PGUM) and (b) by history of hormonal therapy.

There was an association of PGUM and acute ≥ grade 2 GU toxicity (P = .007) but not with the other tested parameters age, HT, PTV, PTV Dmax or bladder V47. Decreased grade ≥ 2 late GU toxicity free survival was associated with higher age (P = .025), absence of HT (P = .016) and higher PGUM (P < .001) but not with the PTV, PTV Dmax or bladder V47 (Table 4; Figure 1). In multiple Cox regression analysis higher PGUM (P < .001) and absence of HT (P = .003) were independently associated with decreased late ≥ grade 2 GU toxicity free survival (Table 5).

**Table 4:** Factors correlating with three year grade ≥ 2 GU toxicity free survival in univariate analysis

**Factor**	**Group**	**No. of Patients**	**% TFS**	No. at Risk*	**p†**
All patients		39	69.2	5	
Age (years)	≤ 69	20	85.0	4	
	> 69	19	52.6	1	.025
Hormonal therapy	no	10	40.0	0	
	yes	29	79.3	5	.016
PGUM (grade)	0–1	29	86.2	4	
	2–3	10	20.0	1	<.001
PTV (cm^3^)	≤ 105	20	80.0	2	
	> 105	19	57.9	3	.128
PTV Dmax (Gy)	≤ 84.3	21	76.2	3	
	> 84.3	18	61.1	2	.199
Bladder V47 (%)	≤ 28	20	75.0	3	
	> 28	19	63.2	2	.437

Abbrevations: TFS = toxicity free survival; PGUM = pre-treatment genitourinary morbidity; PTV = planning target volume; PTV Dmax = maximum dose within the PTV; Bladder V47 = percentage of bladder wall receiving at least 47 Gy * Number at risk at three years; † Log-rank test

**Table 5:** Multiple Cox regression analysis of factors associated with late ≥ grade 2 GU toxicity free survival

**Factor**	**RR**	**CI**	**p**
Age > 69 years versus ≤ 69 years	2.7	0.7–10.9	.143
Hormonal therapy no versus yes	12.4	2.4–64.6	.003
PGUM grade 2-3 versus grade 0–1	29.1	5.4–156.8	<.001

Abbreviation: RR = relative risk; CI = 95% RR confidence intervals; PGUM = pre-treatment genitourinary morbidity.

### PSA nadir

With a median follow-up of 29 months it is too early to report biochemical control rates. However, after treatment 93% (27 out of 29) of patients with and 90% (9 out of 10) of patients without HT reached a PSA nadir value ≤ 0.5 ng/mL. In patients treated by HT, time to nadir was shorter (P < .001) and the nadir value was lower (P = .001) as compared to patients without HT. The median time to nadir was 23.6 months (range, 3.3 – 26.1 months) in patients without and 4.0 months (range, 0.0 – 24.4 months) in patients with HT. The median nadir value was 0.3 ng/mL (range, 0.05 – 5.1 ng/mL) in patients without and 0.07 ng/mL (range, 0.0 – 0.95 ng/mL) in patients with HT.

## Discussion

With the use of more conformal RT techniques, high dose RT of PCA has become the accepted standard of care. Simultaneously, the incidence of treatment related toxicity has become even more important. Here we report the pre-treatment GI and GU morbidity and acute and late toxicity in patients treated by dose escalated high precision RT by IMRT along with organ tracking. The grade 2 acute and maximal late GI toxicity rates were 3% and 8% and late grade 2 GI toxicity decreased to 0% at the end of the follow-up with no grade 3 GI toxicity observed. In face of the grade 2 and 3 PGUM rates of 20% and 5% the observed acute and late grade 2 GU toxicity rates were 56% and 28%. Interestingly the late grade 2 GU toxicity rate dropped to 15% at the end of follow-up being even below the PGUM reported. Acute and late grade 3 GU toxicity was 8% and 3%, respectively. The use of different toxicity scales and RT techniques makes it difficult to compare our data to other studies. Only two studies have reported toxicity rates after PCA treatment with 3D-CRT or IMRT using fiducial markers for position verification [22,23]. Table 6 summarizes the observed toxicity in these studies in comparison with the results of our study. Concerning acute and late GI toxicity our results are excellent, but the maximal GU toxicity rates are higher than those described elsewhere. However, the PGUM in our patient cohort was relatively high and if this is considered, the GU toxicity rates observed in our patients are comparable to those in the literature. The acute GU toxicity was associated with PGUM and the ≥ grade 2 late GU toxicity free survival was significantly decreased in patients with higher age, higher PGUM and in patients who were not treated by HT. The decreased rate of GU late effects in patients undergoing HT is in accordance with pooled data from RTOG trials 85-31, 86-10 and 92-02, where the additional use of HT decreased GU toxicity [24]. The impact of HT on the late GU toxicity might depend on the extent of PGUM, being beneficial especially in patients with obstructive symptoms. The high amount of patients with pre-treatment obstructive symptoms (grade 1 – 3: 49%) might therefore account for the association of HT with decreased GU toxicity.

**Table 6:** Comparison of acute and late GI and GU toxicity rates with the literature

**Study**	**Pts**	**RT**	**Dose (Gy)**	**FU (months)**	**Tox**	**Acute GI grade 2/3**	**Late GI grade 2/3**	**Acute GU grade 2/3**	**Late GU grade 2/3**	**Ref**
Skala et al.	690	3D/IMRT	≤ 79.8	37#	RTOG	n.a.	2.5%/0.7%	n.a.	8.8/0.9	22
Lips et al.	331	IMRT	76	47*	CTC/RTOG^1^	30%/0	9%/1%	47%/3%^2^	21%/4%^2^	23
Current study	39	IMRT	80	29#	CTC	3%/0	8%/0	61%/3%	26%/3%	

Abbreviations: GI = gastrointestinal; GU = genitourinary; Pts = patients; RT = radiotherapy; FU = follow-up; Tox = toxicity score used; Ref = reference; 3D = three-dimensional conformal radiation therapy; IMRT = intensity modulated radiation therapy; RTOG = Radiation Therapy Oncology Group toxicity criteria; n.a. = not applicable; CTC = Common Toxicity Criteria; # median (months); * mean (months); ^1 ^For acute toxicity CTC criteria were used and for late toxicity RTOG criteria. ^2 ^plus 0.3% of patients with grade 4 toxicity.

We are aware of the obvious limitations of our study, being the relatively small number of patients analyzed and the retrospective nature of the study. We also recognize that longer follow-up will be needed to compare the tumor control rates with those after different treatment regiments. Nevertheless, this report provides strong evidence for the need of reporting pre-treatment morbidity rates along with the observed toxicity rates to enhance comparability with other studies.

## Conclusion

GI toxicity rates after dose escalated IMRT and organ tracking are excellent. Acute and late GU toxicity are comparable to other reported series when the pre-treatment GU morbidity is considered.

## Competing interests

The authors declare that they have no competing interests.

## Authors' contributions

Each author had participated sufficiently in the work to take public responsibility for appropriate portions of the content. PG, JV and DMA designed the study. PG, JT and RB performed the statistical analysis. PG, JV, JT, RB, DV, PM, RM, AM and FB collected the data and together with DMA interpreted the data. The manuscript was written by PG, all other authors helped and finally approved the final manuscript.
